# Cardiomyocyte-derived BDNF restricts cardiac fibrosis by decreasing the activity of the TGF-β/Smad2/3 pathway and increasing Smad7 expression

**DOI:** 10.3389/fcell.2026.1786720

**Published:** 2026-04-28

**Authors:** Yu Zhu, Yuanfei Ran, Tingting Fu, Xin Zheng, Yanjun Chen, Chunbao Liang, Yanmei Li, Ruijin Huang, Hui Zhao, Xiudi Pan, Ziqiang Yuan, Qin Pu, Zhaohua Zeng, Shanshan Feng, Xufeng Qi, Luocheng Lv, Lixuan Zhan, Yilin Chen, Dongqing Cai

**Affiliations:** 1 Key Laboratory of Regenerative Medicine, Ministry of Education, Jinan University, Guangzhou, China; 2 Joint Laboratory for Regenerative Medicine, Chinese University of Hong Kong-Jinan University, Guangzhou, China; 3 International Base of Collaboration for Science and Technology (JNU), The Ministry of Science and Technology & Guangdong Province, Guangzhou, China; 4 Department of Developmental & Regenerative Biology, Jinan University Guangzhou, Jinan, China; 5 Department of Neuroanatomy, Institute of Anatomy, Medical Faculty, University of Bonn, Bonn, Germany; 6 Stem cell and Regeneration TRP, School of Biomedical Sciences, Chinese University of Hong Kong, Hong Kong, China; 7 Division of Cardiology, Department of Internal Medicine, The First Affiliated Hospital of Guangzhou Medical University, Guangzhou, China; 8 Department of Medical Oncology, Cancer Institute of New Jersey, Robert Wood Johnson of Medical School, New Brunswick, NB, United States; 9 The First Affiliated Hospital, Key Laboratory of Regenerative Medicine, Ministry of Education, Jinan University, Guangzhou, China; 10 Department of Neurology of the Affiliated Brain Hospital, Guangzhou Medical University, Guangzhou, China; 11 Guangdong Engineering Technology Research Center for Translational Medicine of Mental Disorders, The Affiliated Brain Hospital, Guangzhou Medical University, Guangzhou, China

**Keywords:** BDNF-TrkB pathway, cardiac fibroblasts and cardiac myofibroblasts, cardiac fibrosis, cardiomyocyte-derived BDNF conditional knockout, TGF-β/Smad2/3 pathway

## Abstract

**Background and objective:**

To investigate the role of cardiomyocyte-derived BDNF as an endogenous regulator to decrease cardiac fibrosis, its underlying mechanism and therapeutic potential.

**Methods:**

Single-nuclei RNA sequencing (snRNA-seq), KEGG, Gene Ontology and cell‒cell interaction analyses were performed to identify changes in cardiac cells, cardiac functions and pathways due to the conditional knockout of cardiomyocyte-derived BDNF (cardiomyocyte-BDNF-KO). Protein C-terminal sequencing, qPCR, WB, CCK8 assays, flow cytometry, BDNF-AAV9 treatment and histological staining were performed to investigate the roles of BDNF and the BDNF mimic 7,8-DHF (7,8-DHF) in cardiac fibroblasts cardiac myofibroblasts and cardiac fibrosis, and the cross-inhibition of the TGF-β and BDNF–TrkB-FL pathway.

**Results:**

snRNA-seq and bioinformatics analysis revealed that cardiomyocyte-BDNF-KO significantly increased the percentage of CFs, decreased the number of cardiomyocytes, and increased the activity of the TGF-β pathway in CFs. Functional studies confirmed that compared with those in wild-type hearts, the expression levels of key signaling molecules in the TGF-β pathway in cardiomyocyte-BDNF-KO CFs in the mouse heart were significantly higher. CFs and CMFs expressed the BDNF receptor TrkB-FL but not BDNF, and treatment with BDNF and 7,8-DHF decreased the expression of key signaling molecules in the TGF-β pathway in CFs and CMFs. BDNF inhibited CF and CMF proliferation, inhibited CF activation and transformation into CMFs, promoted CMF apoptosis, the accumulation of cells in S phase of the cell cycle and TrkB-FL phosphorylation in CFs and CMFs, increased Smad7 expression in CMFs, and inhibited the activity of the TGF-β/Smad2/3/α-SMA pathway. 7,8-DHF had the same effects as BDNF, as documented above. Furthermore, BDNF-AAV9 therapy for cardiomyocyte-BDNF-KO hearts increased Smad7 expression and decreased the activity of the TGF-β/Smad2/3 pathway and the expression of fibrotic effectors, which ameliorated cardiac fibrosis.

**Conclusion:**

Cardiomyocyte-derived BDNF acts as an endogenous mediator to restrict cardiac fibrosis by inhibiting CF and CMF proliferation, CF activation and transformation into CMFs, and increasing arrest in S phase of the cell cycle in CFs and CMFs and the apoptosis of CMFs. The BDNF–TrkB-FL pathway cross-inhibits the activity of the TGF-β/Smad2/3/α-SMA pathway and increases the expression of Smad7. BDNF and 7,8-DHF have therapeutic potential for treating cardiac fibrosis.

## Introduction

Cardiac fibrosis is linked to the deterioration of cardiac function, an increased susceptibility to arrhythmia, increased symptomatology, and inferior outcomes of most cardiac diseases (Kato et al., 2015; Gulati et al., 2013). Currently, the effective prevention and treatment of cardiac fibrosis, which is associated with related myocardial pathologies, is still a major challenge in the clinic. Cardiac fibrosis is characterized by the excessive accumulation of extracellular matrix (ECM) components, such as collagens, which are deposited by activated cardiac fibroblasts (CFs), named cardiac myofibroblasts (CMFs), resulting in increased cardiac stiffness and diastolic dysfunction (Henderson et al., 2020; Moreo et al., 2009; Murtha et al., 2019; López et al., 2021). Various stimuli, including inflammation, neurohormonal activation, mechanical stress, aging, and toxic insults, act synergistically to drive cardiac fibrosis, resulting in increased cardiac stiffness and diastolic dysfunction (Moreo et al., 2009; Murtha et al., 2019; López et al., 2021). Current progress in the field suggests that understanding the as yet unknown endogenous mechanism of the myocardium to limit the potential for cardiac fibrosis will be important for the development of curative therapies.

Transforming growth factor beta (TGF-β) is at the core of the regulation of cardiac fibrosis and is implicated in all types of fibrosis (Frangogiannis, 2022; Meng et al., 2016; Khalil et al., 2017; Dobaczewski et al., 2011). Upon activation, the activated TGF-β dimer binds to TGF-β receptor (TβR) II and then TβRI (also known as ALK5). This binding triggers the phosphorylation of the transcription factors Smad2 and Smad3, which, in conjugation with Smad4, then translocate into the fibroblast nucleus to induce the transcription of fibrotic components such as collagens. Activation of the TGF-β pathway promotes the transition of fibroblasts to activated myofibroblasts (Massagué and Xi, 2012). The transformation of CFs into CMFs is the key step in the process of cardiac fibrosis and plays a critical role in the development of fibrosis (Weber et al., 2013). The transformation of CFs into CMFs can be regulated by TGF-β in cardiac fibrosis (Akhmetshina et al., 2012; Villarreal et al., 1992; Piersma et al., 2015). CMF conversion is associated with the acquisition of a matrix synthetic phenotype characterized by high expression levels of both structural collagens and matricellular proteins. Smad3 is critically involved in the activation of a matrix-synthetic cardiac fibroblast phenotype and in the conversion of CFs to CMFs (Dobaczewski et al., 2010). Importantly, in normal cellular physiology, the TGF-β pathway is involved in an endogenous negative regulatory mechanism in which inhibitory Smad7 is an important inducible negative regulator of TGF-β signaling cascades, acting as a competitive inhibitor of receptor-activated Smad activation and nuclear translocation by binding to type I TGF-β receptors (Hanyu et al., 2001) or Smad4 (Hayashi and Sakai, 2012) or by mediating type 1 TGF-β receptor degradation through the recruitment of the ubiquitin ligases Smurf 1 and 2 (Ebisawa et al., 2001), leading to downregulation of TGF-β pathway mediated cardiac fibrosis. In addition, Smad7 expression in CMFs, the activated form of CFs, protects the infarcted myocardium and attenuates adverse remodeling and fibrosis by restraining collagen accumulation and the myofibroblast conversion (Humeres et al., 2022). Therefore, maintaining the TGF-β pathway in the nonactivated state or increasing the expression of Smad7 is crucial for inhibiting the occurrence of cardiac fibrosis. However, the exact endogenous cellular and molecular mechanisms underlying the maintenance of TGF-β pathway activity at the physiological level to prevent the occurrence of cardiac fibrosis in the myocardium is not yet clear. Therefore, the identification of the endogenous mechanisms and pathways that can regulate the activity of the TGF-β pathway in nonprofibrotic scenarios is important for the development of novel strategies to prevent and cure cardiac fibrosis, degenerative cardiomyopathy and heart failure (HF).

Recently, we showed that the ablation of cardiomyocyte-derived BDNF during development causes cardiomyocyte death, myocardial degeneration and cardiac fibrosis, leading to HF in the adult heart. In addition, the ablation of cardiomyocyte-derived BDNF leads to the exacerbation of cardiac dysfunction and poor regeneration after myocardial infarct (MI) in adult hearts (Li et al., 2022). In the present study, we further investigated the possible underlying mechanisms that are affected by the ablation of cardiomyocyte-derived BDNF using single-nuclei RNA sequencing (snRNA-seq) to analyze the transcriptional landscape between cardiomyocyte-derived BDNF knockout (cardiomyocyte-BDNF-KO) hearts and wild-type (WT) hearts. Here, we report for the first time that cardiomyocyte-derived BDNF plays an important role as an endogenous regulator that ameliorates cardiac fibrosis by inhibiting the activity of the TGF-β pathway, CF and CMF proliferation, and CF transformation into CMFs, and increasing the accumulation of CFs and CMFs in S phase of the cell cycle and the apoptosis of CMFs. Importantly, we also revealed for the first time a previously unknown mechanism of the endogenous inhibition of cardiac fibrosis in the myocardium, in which the BDNF–TrkB-FL pathway cross-inhibits the activation of the TGF-β/Smad2/3 pathway and increases the expression of the endogenous negative regulator of the TGF-β pathway, Smad7.

## Materials and methods

### Animals

In the present study, two-month-old male C57BL/6 mice (25 ± 2 g) were used. The mice were allowed to adapt for 1 week before the experiment. The animals were provided food and water *ad libitum*. Cardiomyocyte-BDNF-KO mice (Myh6-Cre^±^-BDNF^flox/flox^) were generated as previously described (Li et al., 2022). Briefly, BDNF^flox/flox^ mice (Stock #004339, Jackson Laboratory) were crossed with B6.FVB-Tg(Myh6-cre)2182Md/J mice (Stock #011038, Jackson Laboratory) to produce heterozygous Myh6-Cre^±^-BDNF^flox/+^ mice, which were then backcrossed to BDNF^flox/flox^ mice to obtain homozygous Myh6-Cre^±^-BDNF^flox/flox^ mice with a mixed background. BDNF^flox/flox^ mice were used as wild-type (WT) controls. Genotyping was performed using the One Step Mouse Genotyping Kit (Vazyme, PD101-01) with the primers previously reported. Animal care, surgery and handling procedures were approved by the Jinan University Animal Care Committee (Approval No. IACUC-2023110106).

### Generation of snRNA-seq datasets from cardiomyocyte-BDNF-KO mouse hearts

Whole hearts from male WT and male cardiomyocyte-BDNF-KO mice were minced and cryopreserved. Nuclei were isolated using a Shbio Nucleus Isolation Kit (SHBIO, #52009–10). Nuclei were counted with a cell counter (Countess 3, Thermo Fisher). Nuclei were immediately loaded onto a Chromium Single-Cell Processor (10X Genomics) for the barcoding of RNA from single nuclei using a Chromium Single-Cell 3′Library and Gel Bead Kit v3 (10X Genomics). All the libraries were generated with tissues from 8-week-old mice. Approximately 20,000 nuclei from each mouse heart sample were used to generate each mouse heart library.

### Clustering of cells by gene expression pattern, assignment of the cell type identity, and determination of the cell type distribution

Sequencing reads, including intronic reads, were processed using Cell Ranger version 5.0.0 and the mm10-1.2.0 reference transcriptome. Next, gene‒barcode matrices were generated for each individual sample by counting UMIs and filtering non-cell-associated barcodes. Finally, a gene‒barcode matrix containing the barcoded cells and gene expression counts was generated. This output was then imported into the Seurat (v5.1.0) R toolkit for quality control and a downstream analysis of our single-cell RNAseq data (Hao et al., 2024). A total of 12,965 nuclei from the wild-type (WT) sample and 15,979 nuclei from the cardiomyocyte-BDNF-KO sample were acquired for analysis. For gene-level quality control, genes that were detected in fewer than 3 cells and genes that were mapped to the mitochondria and hemoglobin were removed. Additionally, cells with more than 30,000 transcripts (nCount_RNA) or fewer than 200 and more than 3,000 genes (nFeature_RNA) were removed. A total of 27,386 (WT: 12,545; KO: 14,841) nuclei were ultimately used for downstream analyses.

For normalization, the top 2,000 highly variable genes were used, and scaling and linear dimension reduction (PCA) were performed on a merged dataset using the R package Seurat (Hao et al., 2024). The ‘integrated.Harmony’ parameter was used to integrate the data from WT and cardiomyocyte-BDNF-KO hearts in Seurat and to enable the identification of shared cell types across cells in WT and cardiomyocyte-BDNF-KO hearts. Next, UMAP was performed using Seurat, in which the nearest neighbor and clustering functions were set using the first 15 principal components from these data. The clustering resolution was set to 0.4 and informed by manual cell type annotations. The expression of known cell-specific gene markers for different individual cell types was used to identify major cell types (Marín-Sedeño et al., 2021; Kelly, 2023; Simonson et al., 2023).

### Differential gene expression analysis

Differentially expressed genes (DEGs; each cell type or differentially expressed genes between each cell cluster) between WT heart and cardiomyocyte-BDNF-KO heart were determined using MAST, which applies a generalized linear model framework that uses the proportion of genes expressed in a single cell as a covariate to account for both technical and biological sources of variation (Hao et al., 2024). For the MAST analysis, FindAllMarkers and FindMarkers were used to identify DEGs with the parameters test. use = Wilcox, min. pct = 0.25, logfc. threshold = 0.25, only. positive = TRUE to clarify the signature genes of each cell group, whereas FALSE was used to identify the DEGs between multiple cell groups. Genes with an adjusted P value < 0.05 were considered significant DEGs (Nee et al., 2024; Wang et al., 2020).

### GO and KEGG enrichment analyses

Gene Ontology (GO) and Kyoto Encyclopedia of Genes and Genomes (KEGG) enrichment analyses were performed on each set of DEGs within each cell group and DEGs across multiple cell groups. This analysis was conducted using the compareCluster function of the clusterProfiler package version 4.12.0 (Wu et al., 2021).

In the DEG enrichment analysis between cardiomyocyte-BDNF-KO hearts and WT hearts, the cardiac structure- and cardiac pathophysiology related GO or KEGG terms which are involved in top 20 (listed in ascending order of the q value) are shown in the results.

### Cell‒cell interaction analysis

The Seurat objects from the WT and cardiomyocyte-specific BDNF-KO snRNA-seq datasets were normalized and analyzed using CellChat 2.0, and the ligand‒receptor interaction database included in CellChat was used for the analysis (Jin et al., 2025). Ligand–receptor interaction databases were integrated with single-cell data. Cell populations with fewer than 30 cells and ligand‒receptor pairs expressed in less than 10% of cells within a population were filtered out prior to the inference of intercellular communication network. The resulting networks were visualized and explored using the built-in tools of CellChat (Jin et al., 2025).

### Cell culture and treatment

Hearts were harvested from anesthetized mice, sterilized in 70% ethanol, and minced in ice-cold PBS to isolate the CFs. The tissue fragments were pooled and digested with collagenase P/trypsin (11249002001; Roche and T819002; Macklin) in DMEM at 37 °C with agitation (flexible shaking). Digestions were repeated until tissue dissolution occurred. The pooled cell suspension was filtered, centrifuged, and plated in complete DMEM (10% FBS, 1% P/S; 11875119 and 10099141c; Gibco). After 1 h of incubation, the medium was replaced to remove nonadherent cells. Adherent CFs were cultured with complete DMEM at 37 °C with 5% CO_2_ in a 95% air incubator to 80%–90% confluence and passaged at a 1:2 ratio. Cells from passages 2–3 were used for the experiments. For the preparation of CMFs, the CFs were isolated and cultured as described above. Then, 10 ng/mL TGF-β1 (HY-P7117, MCE) was added to cultured CFs to induce CMF differentiation for 24 h. Anti-α-SMA (ab5694; Abcam) immunofluorescence staining was performed to confirm successful CMF transformation. In the present study, only prepared CMFs at passage 3 or lower were used for experiments. Both prepared CFs and CMFs were treated with BDNF (HY-P700158AF, MCE; 50 ng/mL, 100 ng/mL and 200 ng/mL) or 7,8-DHF (HY-W013372, MCE; 25 μM, 50 μM, 100 μM, 150 μM and 200 μM) for 24 h and then analyzed. In the present study, 100 ng/mL BDNF and 150 μM BDNF mimic 7,8-DHF were identified as suitable doses. The studies were conducted using three animals and at least three repeated experiments for samples from each individual animal.

### Immunofluorescence staining

CFs were seeded and cultured on glass coverslips treated with lysine in 24-well plates at 37 °C with 5% CO_2_ in a 95% air incubator. After 24 h, the cells were fixed with 4% paraformaldehyde for 15 min at room temperature, washed with PBS, and then permeabilized and blocked with 5% bovine serum albumin (BSA) for 20 min at room temperature. The samples were then incubated overnight at 4 °C with primary antibodies against vimentin (mouse monoclonal; sc-373717; Santa Cruz) and TrkB (rabbit monoclonal; GB11295; Servicebio). Afterward, the samples were incubated with the following fluorescent secondary antibodies: goat anti-mouse IgG conjugated to Alexa Fluor 488 (RGAM002, Proteintech) and goat anti-rabbit IgG conjugated to Alexa Fluor 555 (RGAR003, Proteintech). Nuclei were stained with DAPI (E607303; Sangon). Finally, the coverslips were mounted, and images were captured using a fluorescence microscope.

### Quantitative real-time PCR (qPCR)

Total RNA was extracted from cells using a Cell RNA Rapid Extraction Kit (400–100; Goonie) according to the manufacturer’s instructions. Subsequently, 1 µg of DNase-free RNA was reverse transcribed using Hifair® AdvanceFast 1st Strand cDNA Synthesis SuperMix (11156ES60, Yeasen). Quantitative RT‒PCR (RT‒qPCR) was performed using the fluorescent dye SYBR Green (B110031; Sangon Biotech). The primers were designed using Primer Blast (spanning exons and introns when possible). The details of the primer sequences are shown in Supplementary Table S1. Ct values were measured using a CFX96 instrument (Bio-Rad, United States). The relative fold change in gene expression was calculated with the ΔΔCT method. The expression of GAPDH was used as an endogenous control for normalization. For the quantitative analysis of the RT‒qPCR results, the expression levels in the control group were set to 1 (baseline), and the relative expression levels in the experimental groups were calculated as the fold changes. The results were obtained from at least three independent experiments.

### Western blot

Heart tissues, treated CFs and CMFs were lysed in RIPA buffer (P0013C; Beyotime) supplemented with a protease inhibitor cocktail (W2200S; CWBIO). The total protein concentration was determined using a BCA protein assay kit (P0010; Beyotime). Protein samples were denatured in 5× SDS loading buffer at 95 °C for 10 min, separated on 12% SDS-PAGE gels, and transferred onto PVDF membranes (162–0177; Bio-Rad). After blocking with 5% nonfat milk in TBST, the membranes were incubated overnight at 4 °C with the following primary antibodies: rabbit anti-TrkB (ab18987; Abcam; 1:1000), mouse anti-BDNF (ab203573; Abcam; 1:1000), mouse anti-GAPDH (60004-1-Ig; Proteintech; 1:5000), rabbit anti-TrkB phospho Y705 (ab229908; Abcam; 1:1000), rabbit anti-Smad2/3 (GB111844; Servicebio; 1:1000), rabbit anti-phospho-Smad2 (Ser465/467)/Smad3 (Ser423/425) (#8828; Cell Signaling Technology; 1:1000), mouse anti-Smad7 (68594-1-Ig; Proteintech; 1:5000), rabbit anti-TGF-β (ab179695; Abcam; 1:1000), rabbit anti-collagen I (A16891; ABclonal; 1:1000), and rabbit anti-α-SMA (ab5694; Abcam; 1:1000). The membranes were subsequently washed with TBST and incubated with appropriate horseradish peroxidase (HRP)-conjugated secondary antibodies. The protein bands were visualized using an enhanced chemiluminescence system and imaged with a chemiluminescence reader (GeneGnome HR; Synoptics). All experimental results were obtained from three independent replicates and are presented as fold changes relative to the control group.

### LC–MS/MS analysis of the C-termini of peptides

The protein bands of interest were excised from the SDS‒PAGE gels and subjected to standard procedures for gel digestion, including destaining, reduction, alkylation and tryptic digestion. The resulting peptides were analyzed using LC‒MS/MS (Easy-nLC 1200 and Q Exactive Hybrid Quadrupole-Orbitrap Mass Spectrometer, Thermo Fisher Scientific) with a C18 column coupled to a mass spectrometer operating in data-dependent acquisition mode. The analyzed data were searched against a target database (UniProtKB) using Byonic software (V4.2.4) to identify the C-termini of the analyzed peptides. The sequencing and related analyses were performed by Biotech-Pack Company (Beijing, China).

#### Cell viability and proliferation assays

Cell viability and proliferation were measured using a CCK-8 assay kit (cat. no. C0041; Beyotime) according to the manufacturer’s instructions. Briefly, both prepared CFs and CMFs were treated with BDNF (50 ng/mL, 100 ng/mL and 200 ng/mL) or 7,8-DHF (25 μM, 50 μM, 100 μM, 150 μM and 200 µM) for 24 h. The untreated group was used as a control. Ten microliters of CCK-8 solution were added to each well (100 µL of medium) and incubated for 1 h at 37 °C with 5% CO_2_ in a 95% air incubator. The absorbance was then measured at 450 nm using a microplate reader (Synergy4, BioTek). Based on the results, 100 ng/mL BDNF and 150 µM 7,8-DHF, the BDNF mimic, were identified as suitable doses for subsequent experiments. The studies were conducted using three animals and at least three repeated experiments for samples from each individual animal.

Cell proliferation was further assessed at the single-cell level using the BeyoClick EdU Cell Proliferation Kit with AF488 (C0071S; Beyotime) according to the manufacturer’s instructions. Briefly, after treating CFs and CMFs with BDNF (100 ng/mL) or 7,8-DHF (150 µM) for 24h, followed by incubation with 10 µM EdU for 2 h at 37 °C. Adherent cells were then trypsinized, resuspended in culture medium, and fixed with 4% paraformaldehyde. After permeabilization with 0.3% Triton X-100, the click reaction was performed by incubating the cells with a reaction mixture containing click reaction buffer, CuSO_4_, Azide 488 and click additive solution for 30 min at room temperature in the dark. The cells were subsequently washed and resuspended in PBS for flow cytometric analysis. Unlabeled cells were used to establish voltage settings and gating parameters. AF488 green fluorescence (Ex/Em ∼495/519 nm) was detected using a flow cytometer (Cytoflex; Beckman Coulter), and the percentage of EdU-positive cells within the total cell population was analyzed to quantify the proportion of proliferating cells.

### Flow cytometry analysis of apoptosis

CFs and CMFs were cultured in CF and CMF culture media as described above at 37 °C in a 5% CO_2_ and 95% air incubator. The effects of BDNF (100 ng/mL), the BDNF mimic 7,8-DHF (150 µM), and a vehicle control were observed. After 24 h of treatment, an Annexin V-AF647/PI kit (100–102; Gooine) and subsequent flow cytometry analysis were used to investigate the apoptosis of treated CFs and CMFs according to the manufacturer’s instructions. Briefly, the treated CFs (5 × 10^5^) and CMFs (5 × 10^5^) were washed with cold PBS (pH = 7.4), resuspended in 100 µL of binding buffer and stained with 5 µL of Alexa Fluor 647-conjugated Annexin V. After a 5-min incubation in the dark, 10 µL of PI and 400 µL of binding buffer were added. Finally, the cells were analyzed using a flow cytometer (Cytoflex; Beckman Coulter). Early and late apoptotic cells were examined by plotting fluorescence 2 (FL2 for PI) versus fluorescence 1 (FL1 for Annexin V). A total of 10,000 events were collected and analyzed for each sample. The studies were conducted using three animals and at least three repeated experiments for samples from each individual animal.

#### Cellular senescence assay

Cellular senescence was detected using a senescence-associated β-galactosidase (SA-β-gal) staining kit (C0602; Beyotime, China). CFs or CMFs grown in 6-well plates were fixed and incubated with SA-β-gal staining solution overnight at 37 °C under CO_2_-free conditions. The next day, the cells were observed and imaged using a light microscope.

### Cell cycle analysis

Cell cycle distribution was analyzed using PI/RNase Staining Buffer (40301ES50, YEASEN) following treatment of CFs and CMFs with BDNF (100 ng/mL) or the BDNF mimic 7,8-DHF (150 µM) for 24 h. The cells were collected, washed with PBS (pH = 7.4), and centrifuged at 1600 rpm for 4 min. After removal of the supernatant, cells were fixed in cold 70% ethanol at −20 °C overnight. The fixed cells were then centrifuged at 1600 rpm for 4 min, washed with PBS, and resuspended in 0.5 mL PI/RNase staining buffer. After incubated at room temperature for 15 min, smaple were analyzed by flow cytometry (Cytoflex; Beckman Coulter). Cell cycle distribution (G0/G1, S, and G2/M phases) was quantified using FlowJo software. CFs and CMFs without PI staining were included as unstained controls. All experiments were performed in triplicate and repeated at least three independent times.

#### Treatment of cardiomyocyte-BDNF-KO mice with BDNF-AAV9 or 7,8-DHF

Two-month-old cardiomyocyte-BDNF-KO mice received BDNF-AAV9 (10^12^ viral genomes) via a single tail vein injection (100 μL). The control groups included cardiomyocyte-BDNF-KO mice injected with NC-AAV9 and wild-type (WT) mice (n = 5 mice per group). The BDNF-AAV9 and NC-AAV9 vectors were provided by HANBIO (Shanghai, China). Hearts were collected 16 weeks after the injection.

For 7,8-DHF treatment, two-month-old cardiomyocyte-BDNF-KO mice received daily intraperitoneal injections of 7,8-DHF (5 mg/kg/day in 0.1 mL per 10 g body weight) for 6 weeks. The control groups included untreated cardiomyocyte-BDNF-KO mice and untreated, age-matched WT mice (n = 4 mice per group). 7,8-DHF was dissolved in DMSO and diluted in saline, with a final DMSO concentration less than 10% (v/v). Hearts were harvested after the 6-week treatment period.

### Masson’s trichrome staining

Masson’s trichrome staining was performed using a commercial kit (G1346; Solarbio, China) according to the manufacturer’s instructions. Briefly, paraffin-embedded heart sections were deparaffinized and rehydrated through a graded series of ethanol solutions to distilled water. The sections were then stained with Weigert’s iron hematoxylin for 10 min, differentiated in acid differentiation solution, and blued in Masson’s bluing solution. After staining with Ponceau S acid fuchsin solution for 10 min, the sections were treated with a phosphomolybdic acid solution and counterstained with aniline blue. Special attention was given to the aniline blue staining time to prevent overstaining. Finally, the sections were dehydrated through an ethanol series, cleared in xylene, and mounted with neutral balsam.

### Statistical analysis

All the statistical analyses were performed using R (4.4.0) and GraphPad Prism software (version 10). All the statistical results are presented as the means ± SEMs. Student’s t-test was used for comparisons of two groups. One-way ANOVA was used for comparisons of three groups. p < 0.05 was considered to indicate statistical significance (*p < 0.05, **p < 0.01, ***p < 0.001, and ****p < 0.001).

## Results

### Cardiomyocyte-BDNF-KO significantly increases the percentage of CFs and decreases the percentage of cardiomyocytes

**FIGURE 1:**
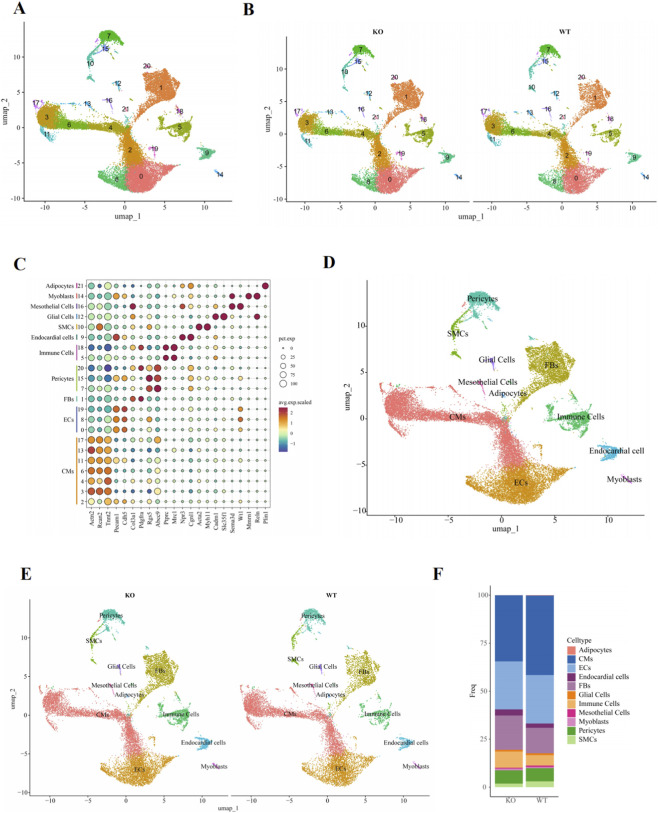
Overview of the cardiac cell composition in cardiomyocyte-BDNF-KO hearts and WT hearts. **(A)** UMAP reduction plot of the full dataset, which was labeled by unsupervised SNN clustering at a resolution of 0.4. **(B)** UMAP dimensionality reduction plot of cardiomyocyte-BDNF-KO hearts and WT hearts grouped by sample origin. **(C)** Dot plot of marker gene expression in cardiac cells. **(D)** UMAP dimensionality reduction plot after semisupervised cell type annotations. **(E)** UMAP dimensionality reduction plot of annotated cell types in cardiomyocyte-BDNF-KO hearts and WT hearts grouped by sample origin. **(F)** Proportions of annotated cardiac cells in cardiomyocyte-BDNF-KO hearts and WT hearts.

Cardiomyocyte-BDNF-KO and WT mice were used to generate the snRNA-seq dataset. We performed cell- and gene-level quality control, followed by sample integration and graph-based clustering, as described in the Materials and Methods, to identify the major cell types present in our data (Hao et al., 2024). The integrated dataset, consisting of the transcriptomes from 27,386 nuclei, revealed a total of 22 distinct clusters, represented by the uniform manifold approximation and projection (UMAP) plot shown in Figures 1A,B. The 22 clusters represented 11 major cell types found in the heart, which were manually annotated using literature-derived annotations with the most enriched marker genes in each population as follows: cardiomyocytes (Actn2, Rcan2, and Tnnt2), endothelial cells (Pecam1 and Cdh5), fibroblasts (Col3a1 and Pdgfra), pericytes (Rgs5 and Abcc9), immune cells (Ptprc and Mrc1), endocardial cells (Npr3 and Cgnl1), smooth muscle cells (Acta2 and Myh11), glial cells (Cadm1 and Slc35f1), epicardial cells (Wt1 and Sema3d), myoblasts (Mmrn1 and Reln), and adipocytes (Plin1) (Figures 1C,D).

An analysis of the distribution of identified cell types in cardiomyocyte-BDNF-KO and WT hearts revealed that all major cell types were represented by each genotype, and no cardiomyocyte-BDNF-KO-specific cell clusters were present (Figure 1E). Significant differences in the cell type distribution were not observed between genotypes (Figure 1E). The number of nuclei for each cell type and genotype is listed in Supplementary Table S2. An analysis of the proportion of each cell type between cardiomyocyte-BDNF-KO and WT hearts revealed that cardiomyocyte-BDNF-KO hearts presented a significantly increased percentage of fibroblasts (ranked first in terms of the increase; KO vs. WT: 17.90% vs. 13.30%) and decreased percentage of cardiomyocytes (ranked first in terms of the decrease; KO vs. WT: 34.40% vs. 41.42%) (Figure 1F; Supplementary Table S2; the percentage difference compared with that in the WT group was greater than 15%, which was statistically significant, and only the number of cells in a cluster greater than 5% of the total cell number of all clusters was compared). The results revealed that ablation of cardiomyocyte-derived BDNF is not associated with a specific cell population in the heart; however, it significantly increases the number of CFs and decreases the number of cardiomyocytes. These cell type-specific changes also strongly suggested that cardiomyocyte-derived BDNF acts as an endogenous mediator to limit cardiac fibrosis and degenerative myocardiopathy, as in cardiomyocyte-BDNF-KO hearts, the number of CFs increased significantly, and the number of cardiomyocytes decreased significantly. Therefore, the present study focuses on CFs, a key effector cell type of cardiac fibrosis, and the functional role and mechanism of BDNF in CFs and cardiac fibrosis.

### KEGG and GO analyses of CF-specific transcriptomic changes between cardiomyocyte-BDNF-KO hearts and WT hearts reveals that genes related to the regulation of cardiac pathways and cardiac structure and function, such as those related to the TGF-β receptor signaling pathway, Smad binding, extracellular matrix structural constituents and collagen-containing extracellular matrix, are enriched

**FIGURE 2:**
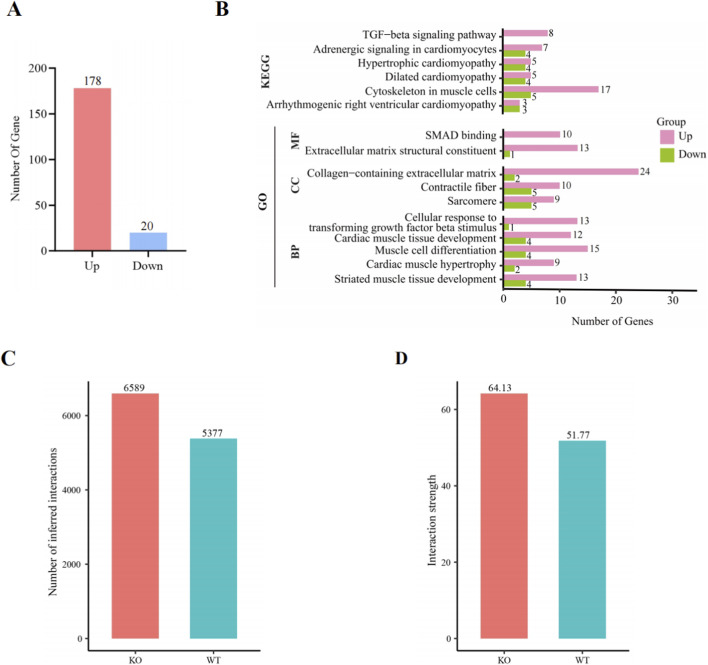
DEGs identified in CFs and KEGG and GO enrichment analyses of the DEGs related to cardiac functions and pathways as well as cell‒cell interactions and an analysis the strength of the interactions between cardiomyocyte-BDNF-KO hearts and WT hearts. **(A)** The number of DEGs (cardiomyocyte-BDNF-KO vs. WT) identified in CFs between cardiomyocyte-BDNF-KO hearts and WT hearts. **(B)** KEGG and GO enrichment analyses of DEGs related to cardiac functions and pathways of CFs. **(C)** An analysis of cell–cell interactions in cardiomyocyte-BDNF-KO hearts and WT hearts revealed that the number of cell–cell interactions was significantly increased in cardiomyocyte-BDNF-KO hearts compared with WT hearts. **(D)** An analysis of cell–cell interactions in cardiomyocyte-BDNF-KO hearts and WT hearts revealed that the strength of cell–cell interactions was significantly increased in cardiomyocyte-BDNF-KO hearts compared with WT hearts.

CFs are among the leading cell types involved in cardiac fibrosis, cardiac remodeling, and pathological changes in dilated and hypertrophic myocardial tissue. Our snRNA-seq analysis of cardiac fibroblasts revealed unique transcriptional changes in cardiomyocyte-BDNF-KO hearts, with 178 upregulated and 20 downregulated DEGs (Figure 2A; Supplementary Table S3). The KEGG pathway analysis indicated that the DEGs were enriched in pathways related to cardiac pathophysiology, including the TGF-β signaling pathway (KEGG: mmu04350), dilated cardiomyopathy (KEGG: mmu05414) and hypertrophic cardiomyopathy (KEGG: mmu05410). Importantly, the expression of all 8 genes included in the enriched TGF-β signaling pathway (KEGG: mmu04350) was upregulated in cardiomyocyte-BDNF-KO hearts compared with that in WT hearts (Figure 2B; Supplementary Tables S4, S6). These findings suggest that the activity of the TGF-β signaling pathway and degenerative cardiomyopathy are increased in the CFs of cardiomyocyte-BDNF-KO hearts.

The GO analysis indicated that the DEGs were enriched in molecular functions related to cardiac structure and cardiac pathophysiology, including Smad binding (GO: 0046332) and extracellular matrix structural constituent (GO: 0005201); cellular components related to cardiac function and structure, including collagen-containing extracellular matrix (GO: 0062023); and biological processes related to cardiac function and processes, including cellular response to TGF-β stimulus (GO: 0071560) and cardiac muscle hypertrophy (GO: 0003300). The DEGs that were included in these identified cardiac structures and cardiac pathophysiological conditions are listed in Supplementary Tables S5, S6. Importantly, the expression of all 10 genes involved in Smad binding (GO: 0046332) was upregulated in cardiomyocyte-BDNF-KO hearts compared with WT hearts. In addition, the number of upregulated genes related to the cellular response to TGF-β stimulus (GO: 0071560), the collagen-containing extracellular matrix (GO: 0062023), and the extracellular matrix structural constituent (GO: 0005201) was 10-fold higher in cardiomyocyte-BDNF-KO hearts than in WT hearts (Figure 2B; Supplementary Table S6). The results of the KEGG and GO analyses suggest that the effects of the ablation of cardiomyocyte-derived BDNF on pathways and cardiac structure and function in CFs involve the activation of the TGF-β signaling pathway, cardiac fibrosis and degenerative myocardiopathy.

### A cell‒cell interaction analysis of the snRNA-seq data reveals a decrease in the activity of the TGF-β pathway in cardiomyocyte-BDNF-KO CFs

**FIGURE 3:**
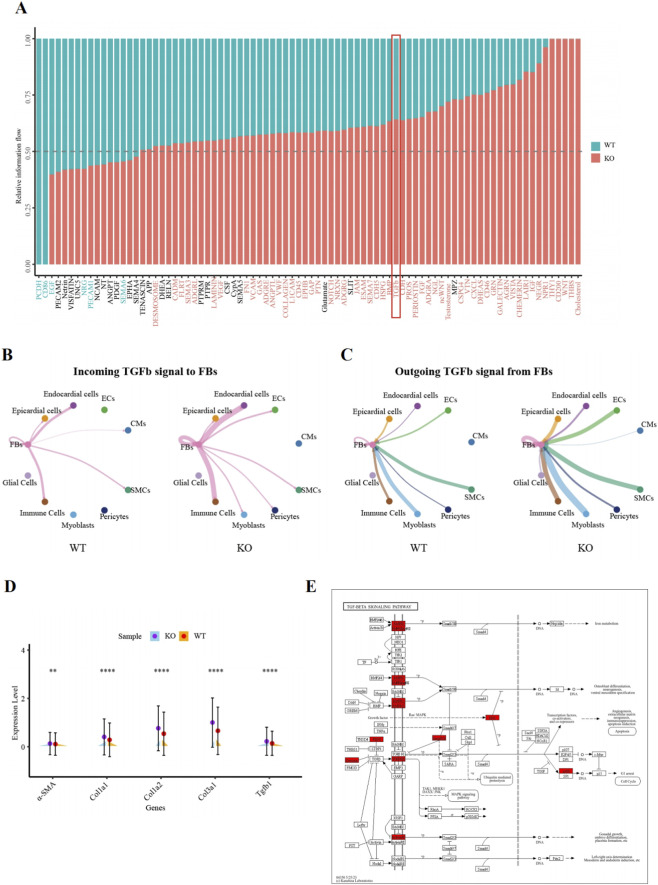
An analysis of cell‒cell interactions by snRNA-seq revealed that cardiomyocyte-BDNF-KO increases the activity of the TGF-β pathway in cardiac fibroblasts. **(A)** CellChat cell‒cell interaction analysis revealed that compared with those in WT hearts, the activity of the TGF-β signaling pathway and 67 other signaling pathways in cardiomyocyte-BDNF-KO hearts increased, whereas the activity of 16 signaling pathways in cardiomyocyte-BDNF-KO hearts decreased. In addition, 5 signaling pathways were identified only in the cardiomyocyte-BDNF-KO hearts but not in the WT hearts, whereas 2 signaling pathways were identified only in the WT hearts but not in the cardiomyocyte-BDNF-KO hearts. **(B)** Analysis of the activity of the TGF-β pathway during interactions between the CFs and other cardiac cells with respect to the function of CFs as ligand-secreting cells in WT hearts and cardiomyocyte-BDNF-KO hearts. **(C)** Activity of the TGF-β pathway during interactions between the CFs and other cardiac cells with respect to the function of CFs as recipients in WT hearts and cardiomyocyte-BDNF-KO hearts. B–C: Compared with those in WT hearts, the strength of the TGF-β pathway in CFs of cardiomyocyte-BDNF-KO hearts was increased regardless of the type of ligand-secreting cell and recipient cell. **(D)** snRNA-seq revealed that the expression of key signaling molecules in the TGFβ pathway (*TGF-β1, α-SMA, Col1a1, Col1a2* and *Col3a1*) in CFs of cardiomyocyte-BDNF-KO hearts was significantly higher than that in CFs of WT mice. **(E)** DEGs identified in the CF snRNA-seq dataset between the cardiomyocyte-BDNF-KO hearts and WT hearts were subjected to a KEGG signaling pathway analysis. Eight genes (*Acvr1, Bmpr2, Dcn, Ep300, Fbn1, Mapk1, Smurf2,* and *Tgfbr2*) involved in the TGF-β signaling pathway were enriched, and their expression levels increased.

Accordingly, cell‒cell interactions between CFs and other cardiac cells were further investigated by comparing the snRNA-seq data from cardiomyocyte-BDNF-KO hearts and WT hearts using CellChat 2.0. In this analysis, only the expression of receptors and ligands in the cells of any type from the different groups >10% was included to identify ligand–receptor interactions among all clusters of cells (Jin et al., 2025). Compared with those in WT hearts, the number and strength of cell‒cell interactions significantly increased (KO vs. WT: number: 6589 vs. 5377; strength: 64.13 vs. 51.77) in cardiomyocyte-BDNF-KO hearts (Figures 2C,D). According to the results of the cell‒cell interaction analysis, 84 signaling pathways were validated in the hearts of cardiomyocyte-BDNF-KO and WT mice. Among them, the activity of the TGF-β signaling pathway and 67 other signaling pathways was increased in the cardiomyocyte-BDNF-KO hearts compared with the WT hearts, whereas the activity of 16 signaling pathways was decreased in the cardiomyocyte-BDNF-KO hearts compared with the WT hearts. In addition, 5 signaling pathways were identified only in the hearts of the cardiomyocyte-BDNF-KO mice but not in those of the WT mice, whereas 2 signaling pathways were identified only in the hearts of the WT mice but not in those of the cardiomyocyte-BDNF-KO mice (Figure 3A).

The TGF-β pathway is a well-established pathway that activates cardiac fibrosis and is involved in all types of fibrosis (Frangogiannis, 2022; Meng et al., 2016; Khalil et al., 2017; Dobaczewski et al., 2011). Therefore, the finding of increased TGF-β pathway activity in the cardiomyocyte-BDNF-KO heart prompted us to further analyze the TGF-β-pathway and interactions between CFs and other cardiac cells through two mechanisms. For CFs functioning as ligand-secreting cells, compared to WT hearts, the strength of the signals transmitted by the TGF-β-pathway from CFs to CFs, endothelial cells, endocardial cells, immune cells, myoblasts and pericytes was increased in the cardiomyocyte-BDNF-KO hearts, whereas the strength of the signals transmitted from CFs to cardiomyocytes was decreased (Figure 3B). For CFs serving as recipients, compared to WT heart, the strength of signals transmitted by the TGF-β-pathway from CFs to CFs as well as from cardiomyocytes, endothelial cells, endocardial cells, epicardial cells, immune cells, myoblasts, smooth muscle cells, pericytes, and glial cells to CFs, was increased in cardiomyocyte-BDNF-KO hearts (Figure 3C). These results suggested that compared cardiomyocyte-BDNF-KO with that in WT hearts, the strength of the activity of the TGF-β pathway in the hearts of cardiomyocyte-BDNF-KO mice was greater, regardless as the ligand-secreting cells and recipient cells.

Accordingly, the expression of key signaling molecules in the TGF-β pathway in CFs was further compared between cardiomyocyte-BDNF-KO hearts and WT hearts using an snRNA-seq dataset. The expression levels of *TGF-β1*, *α-SMA*, *Col1a1*, *Col1a2* and *Col3a1* in the hearts of the cardiomyocyte-BDNF-KO mice were significantly higher than those in the hearts of the WT mice (Figure 3D). Furthermore, the DEGs in the CF snRNA-seq dataset between the cardiomyocyte-BDNF-KO hearts and WT hearts were subjected to a KEGG signaling pathway analysis. Eight genes (*Acvr1, Bmpr2, Dcn, Ep300, Fbn1, Mapk1, Smurf2,* and *Tgfbr2*) involved in the TGF-β signaling pathway were enriched, and their expression levels increased (Figure 3E). These results clearly suggest that cardiomyocyte-BDNF-KO increases the activity of the TGF-β pathway in CFs and promotes interactions between CFs and with other cardiac cells, such as endothelial cells, immune cells, and endocardial cells. Thus, cardiomyocyte-derived BDNF plays an important role as endogenous regulator that maintain the activity of the TGF-β pathway at normal levels, which contributes to the inhibition of cardiac fibrosis in the myocardium.

### CFs express the BDNF receptor TrkB-FL but not BDNF, and BDNF treatment decreases the expression of key signaling molecules in the TGF-β pathway in CFs and CMFs

**FIGURE 4:**
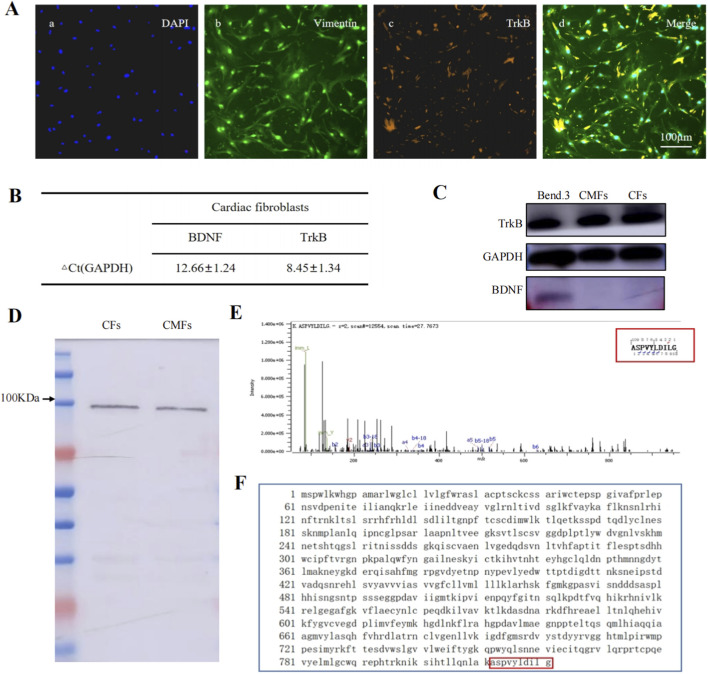
CFs express the BDNF receptor TrkB-FL but not BDNF. **(A)** Anti-Vimentin (marker of CFs) and anti-TrkB (BDNF receptor) double immunofluorescence staining confirmed that the isolated CFs from WT hearts were positive for vimentin and TrkB expression. **(B)** The results of the qPCR analysis revealed that the CFs did not express BDNF but did express TrkB, BDNF receptor. **(C)** WB analysis showing that the CFs from WT hearts and CMFs induced by TGF-β treatment expressed the BDNF receptor TrkB but not BDNF. Bend.3: Brain-derived Endothelial cells.3(Bend.3) cell line, used as a positive control for BDNF and TrkB expression. **(D)** The isolated TrkB bands from the CFs and CMFs used for C-terminal sequencing. **(E)** The 9-amino acid sequence of the C-terminal sequence of the proteins in “D”. **(F)** The amino acid sequence of TrkB-FL. The amino acid sequence of the protein in “E” is 100% identical to the C-terminal 9 amino acids of the protein in “F”. **(D–F)** Protein C-terminal sequencing revealed that CFs express the BDNF full-length receptor TrkB-FL but not the truncated receptor TrkB-T1.

Next, functional studies were performed to investigate the possible effects of BDNF on the regulation of TGF-β pathway activity in CFs and activated CFs, CMFs. We first isolated CFs from WT hearts. Anti-vimentin (marker of CFs) and anti-TrkB (BDNF receptor) double immunofluorescence staining confirmed that the isolated CFs were positive for vimentin and TrkB expression (Figure 4A). qPCR analysis revealed that the CFs did not express BDNF but did express TrkB (Figure 4B). WB analysis further showed that the CFs from WT hearts and CMFs induced by TGF-β treatment expressed the BDNF receptor TrkB but not BDNF. A mouse brain endothelial cell line (Bend.3), which was used as a positive control for TrkB and BDNF expression, also confirmed that the Bend.3 cells were BDNF- and TrkB-positive (Figure 4C). C-terminal protein sequencing revealed that CFs expressed the full-length BDNF receptor TrkB-FL but not the truncated TrkB-T1 (Figures 4D–F). The findings indicate that CFs and CMFs can receive and transduce BDNF signals through the binding of BDNF to their TrkB-FL receptors, although they do not express BDNF. These findings, combined with those of the cell‒cell interaction analysis of snRNA-Seq data from the cardiomyocyte-BDNF-KO hearts and WT hearts, demonstrated that cardiomyocyte-BDNF-KO increased the strength of the activity of the TGF-β pathway in CFs, strongly suggesting that cardiomyocyte-derived BDNF is able to regulate the function of CFs and activated CFs, CMFs by binding to the TrkB-FL receptor on these cells to cross talk and turndown the activity of the TGF-β pathway and limit cardiac fibrosis.

**FIGURE 5:**
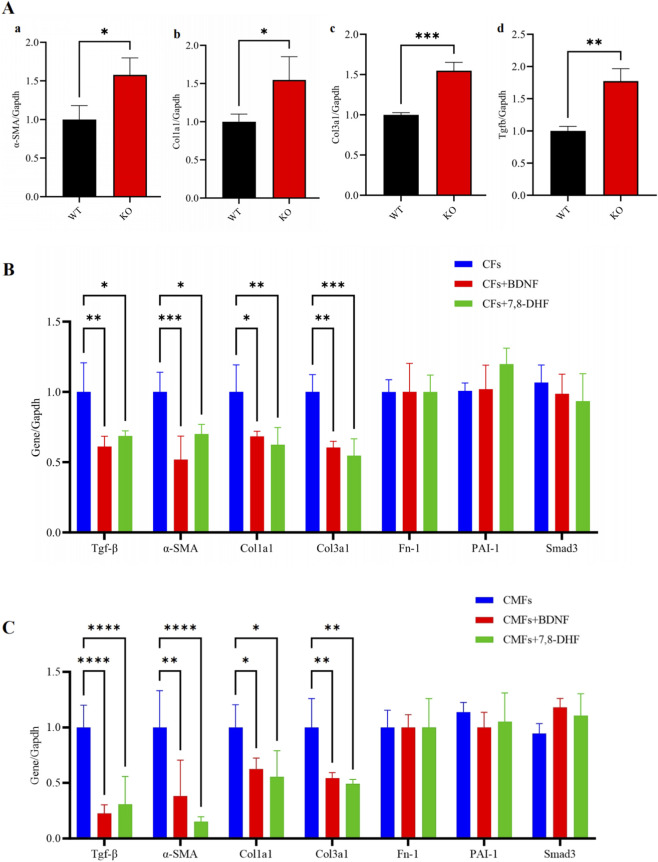
The expression levels of key signaling molecules in the TGF-β pathway in the CFs of cardiomyocyte-BDNF-KO hearts are increased, and BDNF and 7,8-DHF treatments decrease the expression of key signaling molecules in the TGF-β pathway in the CFs and CMFs. **(A)** qPCR analysis revealed that the expression levels of representative key signaling molecules in the TGF-β pathway (*α-SMA*(a)*, Col1a1* (b)*, Col3a1*(c)*, and TGF-β*(d)) in the CFs of cardiomyocyte-BDNF-KO hearts were significantly higher than those in the WT hearts. *: *p* < 0.05. n = 3. **(B)** qPCR analysis revealed that both BDNF treatment and 7,8-DHF treatment decreased the expression levels of key signaling molecules in the TGF-β pathway (*TGF-β, α-SMA, Col1a1 and Col3a1*) in the CFs of WT hearts *in vitro*. *: *p* < 0.05. **: *p* < 0.01. ***: *p* < 0.001. n = 3. **(C)** qPCR analysis showed that both BDNF treatment and 7,8-DHF treatment decreased the expression levels of key signaling molecules in the TGF-β pathway (*TGF-β, α-SMA, Col1a1 and Col3a*) in CMFs *in vitro*. *: *p* < 0.05. **: *p* < 0.01. ***: *p* < 0.001. n = 3.

Therefore, the expression levels of representative key signaling molecules involved in the TGF-β pathway (*α-SMA, Col1a1, Col3a1 and TGF-β*) in the CFs from cardiomyocyte-BDNF-KO hearts and WT hearts were further compared. Our qPCR analysis revealed that the expression levels of the *α-SMA, Col1a1, Col3a1 and TGF-β* genes in the CFs from the hearts of cardiomyocyte-BDNF-KO mice were significantly higher than those in the hearts of WT mice (Figure 5A), which confirmed that the activity of the TGF-β pathway in the CFs of cardiomyocyte-BDNF-KO mice was increased. In addition, we further observed that treatment with both BDNF and a BDNF mimic, 7,8-dihydroxyflavone (7,8-DHF; which can bind with high affinity to the BDNF TrkB receptor), decreased the expression levels of the *TGF-β, α-SMA, Col1a1 and Col3a1* genes in the CFs (Figure 5B) and CMFs (Figure 5C) of WT hearts *in vitro*. Together, these results suggest that cardiomyocyte-derived BDNF and 7,8-DHF can decrease the expression of key signaling molecules in the TGF-β pathway and inhibit the activity of the TGF-β pathway in CFs and CMFs. Therefore, it might function in the regulation of CF proliferation and CF transformation into CMFs to decrease the cardiac fibrosis potential in the myocardium by inhibiting the activity of the TGF-β pathway.

### BDNF inhibits the proliferation of CFs and CMFs and promotes the apoptosis of CMFs and the accumulation of CFs and CMFs in S phase

**FIGURE 6:**
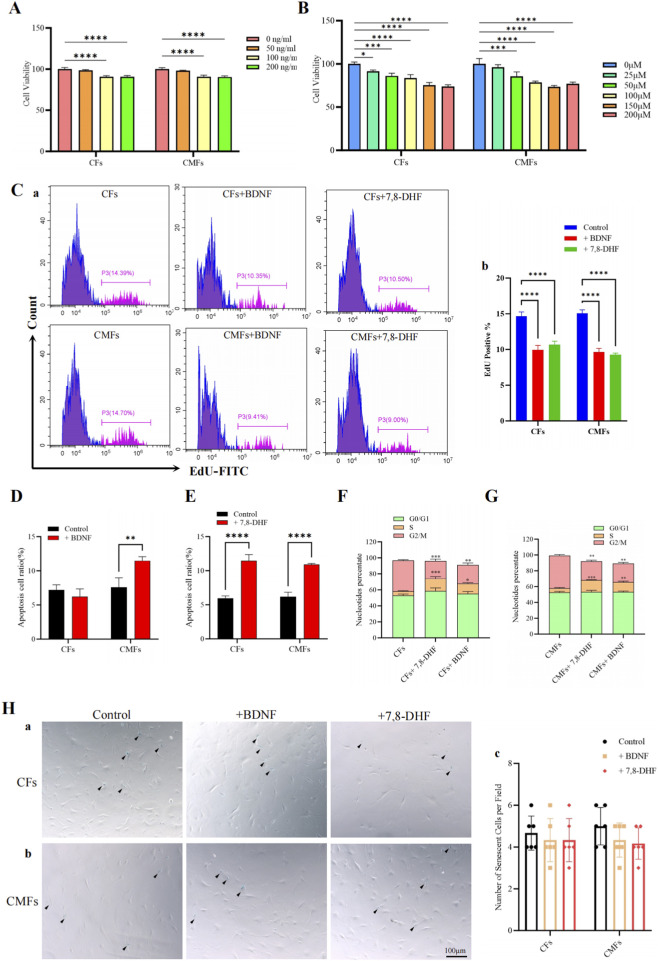
BDNF inhibits the proliferation of CFs and CMFs and promotes the apoptosis of CMFs and the accumulation of CFs and CMFs in S phase but does not induce senescence in CFs or CMFs, and 7,8-DHF has the same effects as BDNF. **(A)** The results of the CCK8 assay showed that BDNF treatment inhibited the proliferation and survival of CFs and CMFs. n = 4. **(B)** The results of the CCK-8 assay revealed that 7,8-DHF treatment inhibited the proliferation and survival of CFs and CMFs. n = 4. A and **(B)** A dose of 100 ng/mL BDNF and 50 μM 7,8-DHF, the BDNF mimic, was suitable for all the following studies in the present study. **(C)** EdU incorporation combining with flow cytometry assays showed that both BDNF and 7,8-DHF treatment inhibited the proliferation of CFs and CMFs. a: Representative images of flow cytometry analysis using EdU incorporation for proliferation in CFs and CMFs treated with or without BDNF and 7,8-DHF. b: Semiquantitative analysis of the data shown in a. n = 3. **(D)** The flow cytometry analysis by using Annexin V-AF647/PI double staining revealed that BDNF treatment promoted the apoptosis of CMFs but not of CFs. n = 3. **(E)** The flow cytometry analysis by using Annexin V-AF647/PI double staining revealed that 7,8-DHF treatment promoted the apoptosis of CMFs and CFs. n = 3. **(F)** PI staining for the cell cycle and flow cytometry revealed that both BDNF and 7,8-DHF treatments resulted in the accumulation of CFs in S phase. **(G)** PI staining for the cell cycle and flow cytometry revealed that both BDNF and 7,8-DHF treatments resulted in the accumulation of CMFs in S phase. n = 3. **(H)** β-Gal staining showed that neither BDNF nor 7,8-DHF treatment increased the number of senescent CFs or CMFs. a: Representative images of CFs treated with BDNF, 7,8-DHF or the control. b: Representative images of CMFs treated with BDNF, 7,8-DHF, or the control. c: Semiquantitative analysis of the data shown in a and b. n = 3. *: *p* < 0.05. **: *p* < 0.01. ***: *p* < 0.001. ****: *p* < 0.0001.

**FIGURE 7:**
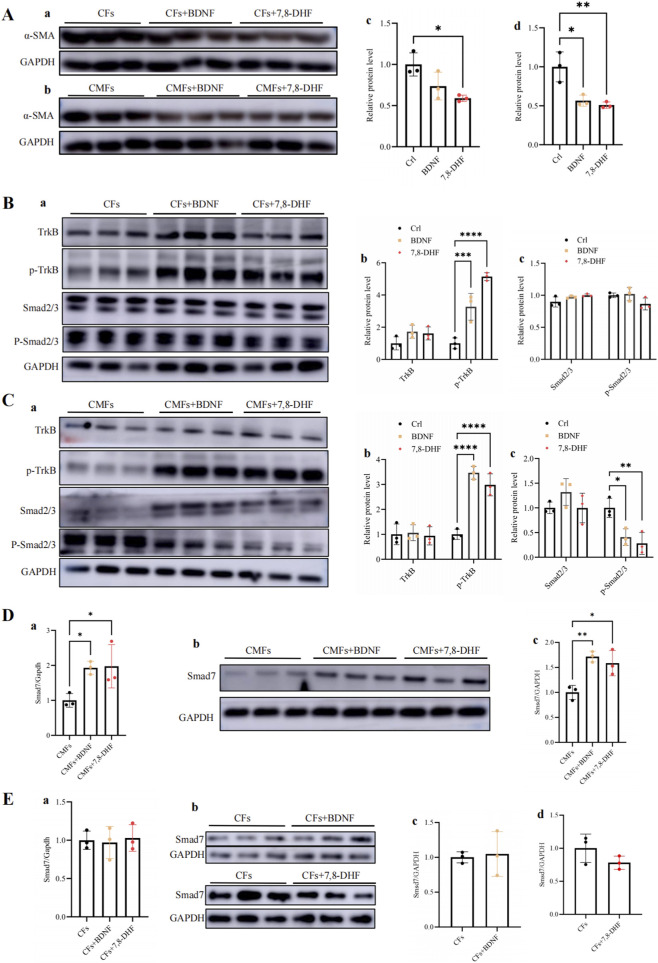
BDNF and 7,8-DHF treatments decreased the expression of α-SMA in CFs and CMFs, promoted the phosphorylation of the TrkB receptor in CFs and CMFs, decreased the phosphorylation of Smad2/3, and increased the expression of Samd7 in CMFs. **(A)** WB analysis revealed that both BDNF treatment and 7,8-DHF treatment decreased the expression of α-SMA, a key protein involved in cardiac fibrosis, in both CFs and CMFs. c: Semiquantitative analysis of the CFs shown in “a”. d: Semiquantitative analysis of the CMFs shown in “b”. **(B)** WB analysis revealed that both BDNF treatment and 7,8-DHF treatment promoted the phosphorylation of the TrkB receptor in CFs but did not change the expression level of the TrkB receptor; moreover, BDNF treatment and 7,8-DHF treatment did not affect the expression or phosphorylation of Smad2/3 in CFs. b: Semiquantitative analysis of the TrkB receptor levels in CFs shown in “a”. c: Semiquantitative analysis of Smad2/3 levels in CFs shown in “a”. **(C)** WB analysis revealed that both BDNF treatment and 7,8-DHF treatment promoted the phosphorylation of the TrkB receptor in CMFs, whereas they decreased the phosphorylation of Smad2/3 in CMFs but did not change the expression level of Smad2/3 in CMFs. b: Semiquantitative analysis of the TrkB receptor levels in CMFs shown in “a”. c: Semiquantitative analysis of “Smad2/3 levels in CMFs shown in “a”. **(D)** qPCR (a) and WB (b and c) analyses confirmed that both BDNF treatment and 7,8-DHF treatment increased the expression level of Smad7 in CMFs. **(E)** qPCR (a) and WB (b and c) analyses confirmed that neither BDNF nor 7,8-DHF treatment altered the expression level of Smad7 in CFs. *: *p* < 0.05. **: *p* < 0.01. ***: *p* < 0.001. ****: *p* < 0.0001. n = 3.

The effects of BDNF and 7,8-DHF on the proliferation, apoptosis and cell cycle of CFs and CMFs were also evaluated. The results of the CCK-8 assay showed that both BDNF (Figure 6A) and 7,8-DHF (Figure 6B) inhibited the proliferation and survival of CFs and CMFs. The results revealed that the suitable doses of BDNF and the BDNF mimic 7,8-DHF were 100 ng/mL and 150 μM, respectively; therefore, these two doses were applied in subsequent studies. Furthermore, EdU incorporation assays (Figure 6C) confirmed that both BDNF and 7,8-DHF treatment inhibited the proliferation of CFs and CMFs. In addition, the flow cytometry analysis by using Annexin V-AF647/PI double staining revealed that BDNF treatment promoted the apoptosis of CMFs but not of CFs (Figure 6D; Supplementary Figure S1A). 7,8-DHF treatment promoted the apoptosis of CMFs and CFs (Figure 6E; Supplementary Figure S1B). Furthermore, the results of the PI staining and flow cytometry assay revealed that both BDNF (Figure 6F; Supplementary Figure S1C) and 7,8-DHF (Figure 6G; Supplementary Figure S1C) treatment resulted in the accumulation of CFs and CMFs in S phase. However, β-gal staining showed that neither BDNF nor 7,8-DHF treatment increased the number of senescent CFs or CMFs (Figure 6H). These results indicate that the BDNF-mediated decrease in CF proliferation is caused mainly by a delay in S phase of the cell cycle. However, the BDNF-mediated decrease in CMF proliferation is caused mainly by a delay in S phase of the cell cycle and the induction of apoptosis. In addition, BDNF is more likely to promote apoptosis in CMFs than in CFs. Thus, BDNF not only inhibits proliferation and cell cycling in CFs but also simultaneously affects the same functions and induces apoptosis in CMFs. This unique feature indicates that BDNF is a natural endogenous mediator that limits the activation of CFs and inhibits their transformation into CMFs to inhibit cardiac fibrosis in the myocardium. 7,8-DHF has effects similar to those of BDNF but more strongly promotes the apoptosis of CFs; therefore, it is suitable for use as a substitute for BDNF.

### BDNF treatment promotes the phosphorylation of the TrkB receptor, increases Smad7 expression, and inhibits TGF-β/Smad2/3/α-SMA activity in the cardiac fibrosis pathway

The molecular mechanism by which the BDNF‒TrkB pathway inhibits the activity of the TGF-β pathway to achieve a decrease in cardiac fibrosis was further investigated. WB analysis revealed that both BDNF treatment and 7,8-DHFtreatment decreased the expression of α-SMA (Figure 7A), a key protein involved in cardiac fibrosis in both CFs and CMFs, and the transformation of CFs into CMFs. These findings suggest that both BDNF and 7,8-DHF decrease cardiac fibrosis activity by decreasing the expression of α-SMA in both CFs and CMFs and inhibiting CF transformation into CMFs. In addition, WB analysis further revealed that in CFs, BDNF and 7,8-DHF treatment promoted only the phosphorylation of the TrkB receptor but not the phosphorylation of Smad2/3 (Figure 7B); however, treatment with both BDNF and the BDNF mimic 7,8-DHF promoted the phosphorylation of the TrkB receptor and decreased phosphorylation of Smad2/3 in CMFs (Figure 7C). These results suggest that BDNF can activate the BDNF–TrkB-FL pathway in both CFs and CMFs. Importantly, with respect to CMFs, the activated BDNF–TrkB-FL pathway can cross-regulate the inhibition of the cardiac fibrosis-mediated pathway, the TGF-β pathway via decreased phosphorylation of Smad2/3, as the TGF-β/Smad2/3 pathway is well established as a key pathway involved in cardiac fibrosis and the phosphorylation of Smad2/3 is a key marker of the activation of the TGF-β/Samd2/3 pathway (Stockis et al., 2017; Budi et al., 2021). Therefore, the results suggest that BDNF-induced activation of TrkB signaling can cross-inhibit the TGF-β/Smad2/3 pathway in activated CFs, CMFs and subsequently inhibit the activity of cardiac fibrosis.

Smad7 is a well-known negative regulator of the TGF-β/Smad2/3 pathway (Humeres et al., 2022). Smad7 is associated with activated TGF-β type I receptors (Ebisawa et al., 2001; Humeres et al., 2022), which antagonize TGF-β signaling through multiple mechanisms, including interfering with R-Smad recruitment, promoting receptor dephosphorylation, recruiting E3 ubiquitin ligases to induce receptor degradation, and blocking the functional Smad complex from interacting with DNA in the nucleus (Yan and Chen, 2011). Therefore, we further investigated the possible effect of BDNF on Smad7 expression in CMFs and CFs. Our qPCR and WB analyses confirmed that both BDNF treatment and 7,8-DHF treatment increased Smad7 expression in CMFs (Figure 7D). However, the results of the qPCR and WB analyses revealed that neither BDNF nor 7,8-DHF treatment increased Smad7 expression in the CFs (Figure 7E). These results further suggest that BDNF treatment increases Smad7 expression and subsequently antagonizes TGF-β signaling, which is another mechanism underlying the inhibition of the TGF-β/Smad2/3 pathway by BDNF–TrkB-FL signaling in CMFs. However, in CFs, BDNF treatment failed to increase the inhibition of the TGF-β signaling pathway by Smad7. Taken together, the above findings strongly support that the BDNF–TrkB-FL pathway cross-inhibits the activity of the TGF-β/Smad2/3/α-SMA-related cardiac fibrosis pathway by decreasing the phosphorylation of Smad2/3 and increasing Smad7 expression in CMFs. Furthermore, the effects of 7,8-DHF are similar to those of BDNF.

### BDNF-AAV9-mediated ectopic BDNF gene therapy for cardiomyocyte-BDNF-KO hearts *in vivo* increases Smad7 expression and decreases the activity of the TGF-β/Smad2/3 pathway and the expression levels of fibrotic markers and improves cardiac fibrosis

**FIGURE 8:**
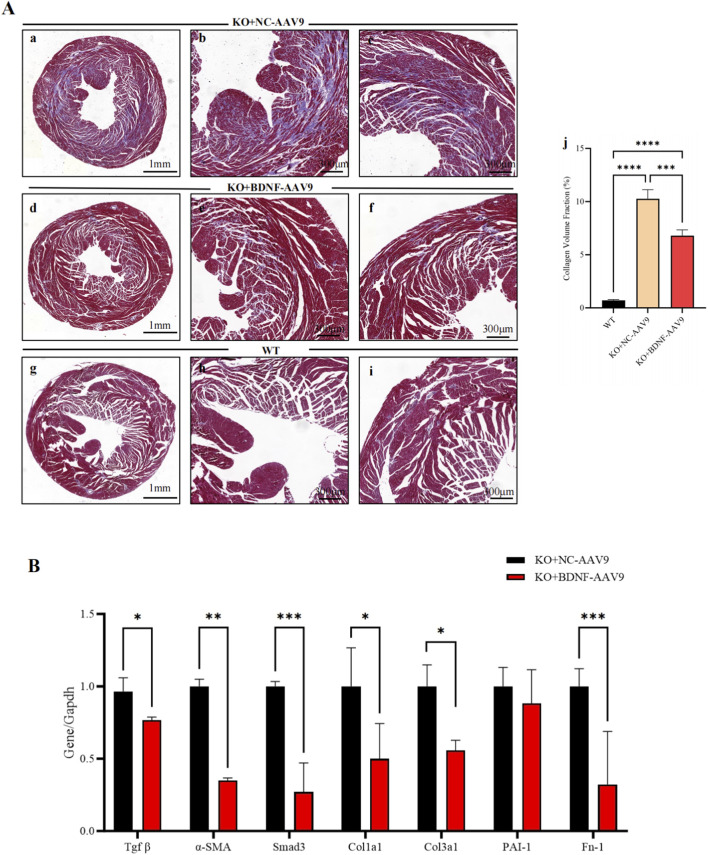
BDNF-AAV9-mediated ectopic BDNF gene therapy decreased the expression levels of cardiac fibrosis effectors and ameliorated cardiac fibrosis in cardiomyocyte-BDNF-KO hearts *in vivo*. **(A)** Masson’s trichrome staining showed that after treatment for 16 weeks *in vivo*, the fibrotic area of BDNF-AAV9-treated hearts was still larger than that of WT hearts but was significantly smaller than that of NC-AAV9-treated cardiomyocyte-BDNF-KO control hearts. a–c: Cardiomyocyte-BDNF-KO hearts treated with NC-AAV9. d–f: Cardiomyocyte-BDNF-KO hearts treated with BDNF-AAV9. g–i: WT hearts. b and c: Magnified images of a. e–f: Magnified images of d. h–i: Magnified images of g. j: Semiquantitative analysis of fibrosis area (blue) of cardiomyocyte-BDNF-KO hearts treated with NC-AAV9, cardiomyocyte-BDNF-KO hearts treated with BDNF-AAV9 and WT hearts. n = 5. **(B)** qPCR analysis revealed that compared with those in NC-AAV9-treated cardiomyocyte-BDNF-KO control hearts, BDNF-AAV9 treatment significantly decreased the expression of genes related to TGF-β/Smad2/3-mediated cardiac fibrosis (*TGF-β, α-SMA, Smad3, Cola1, Col3a1* and *Fn-1*) in cardiomyocyte-BDNF-KO hearts. n = 5. *: *p* < 0.05. **: *p* < 0.01. ***: *p* < 0.001. ****: *p* < 0.0001.

**FIGURE 9:**
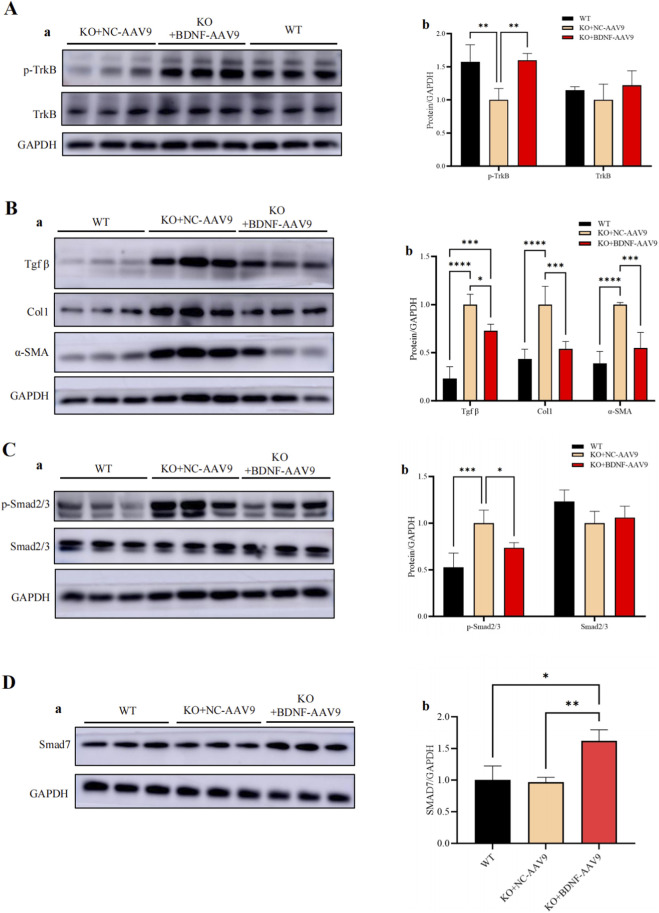
BDNF-AAV9-mediated ectopic BDNF gene therapy for cardiomyocyte-BDNF-KO hearts *in vivo* decreases the activity of the TGF-β/Smad2/3 pathway and increases Smad7 expression. **(A)** WB analysis revealed that the phosphorylation of the TrkB receptor in BDNF-AAV9-treated hearts was significantly higher than that in NC-AAV9-treated hearts. a: Representative images of WBs. b: Semiquantitative analysis of the data shown in “a”. **(B)** WB analysis showed that the protein expression levels of TGF-β, Col1, and α-SMA (key effectors of cardiac fibrosis mediated by the TGF-β/Smad2/3 pathway) in BDNF-AAV9-treated cardiomyocyte-BDNF-KO hearts were significantly lower than those in NC-AAV9-treated cardiomyocyte-BDNF-KO hearts. a: Representative images of WBs. b: Semiquantitative analysis of the data shown in “a”. **(C)** WB analysis showing the levels of phosphorylated Smad2/3 in BDNF-AAV9-treated cardiomyocyte-BDNF-KO hearts and WT hearts were significantly lower than those in NC-AAV9-treated cardiomyocyte-BDNF-KO hearts. a: Representative images of WBs. b: Semiquantitative analysis of the data shown in “a”. **(D)** WB analysis showed that the Smad7 level in BDNF-AAV9-treated cardiomyocyte-BDNF-KO hearts was significantly higher than that in NC-AAV9-treated cardiomyocyte-BDNF-KO hearts and WT hearts, whereas Smad7 expression in NC-AAV9-treated cardiomyocyte-BDNF-KO hearts was similar to that in WT hearts. a: Representative images of WBs. b: Semiquantitative analysis of the data shown in “a”. n = 3. *: *p* < 0.05. **: *p* < 0.01. ***: *p* < 0.001. ****: *p* < 0.0001.

The effect of BDNF expression on rescuing cardiomyocyte-BDNF-KO hearts was investigated to investigate whether restoring BDNF expression levels in the myocardium could reverse the upregulation of the TGF-β/Smad2/3 pathway and fibrotic parameters and attenuate the degree of cardiac fibrosis observed in cardiomyocyte-BDNF-KO hearts. A high-myocardium-affinity AAV9-mediated ectopic BDNF gene expression vector was used to induce BDNF overexpression in the myocardium of cardiomyocyte-BDNF-KO hearts for 16 weeks and investigate the long-term therapeutic effect. Sixteen weeks after BDNF-AAV9 treatment, the area of cardiac fibrosis in BDNF-AAV9-treated cardiomyocyte-BDNF-KO hearts was still higher than that in WT hearts; however, it was significantly lower than that in NC-AAV9-treated cardiomyocyte-BDNF-KO hearts (Figure 8A). These findings revealed that restoring BDNF expression levels in the myocardium attenuated cardiac fibrosis mediated by the loss of cardiomyocyte-derived BDNF. In addition, the qPCR analysis revealed that compared to NC-AAV9-treated cardiomyocyte-BDNF-KO hearts, the expression of genes related to the TGF-β/Smad2/3 pathway and cardiac fibrosis (*TGF-β, α-SMA, Smad3, Cola1, Col3a1,* and *Fn-1*) was significantly decreased in cardiomyocyte-BDNF-KO hearts (Figure 8B). Furthermore, WB analysis showed that the phosphorylation level of the TrkB receptor in BDNF-AAV9-treated cardiomyocyte-BDNF-KO hearts was similar to that in WT control hearts but significantly higher than that in NC-AAV9-treated cardiomyocyte-BDNF-KO hearts (Figure 9A). In addition, WB analysis revealed that the protein expression levels of *TGF-β, Col1,* and *α-SMA* (indicators of cardiac fibrosis mediated by the TGF-β/Smad2/3 pathway) in NC-AAV9-treated cardiomyocyte-BDNF-KO hearts were significantly higher than those in WT hearts, whereas their expression levels in BDNF-AAV9-treated cardiomyocyte-BDNF-KO hearts were significantly lower than those in NC-AAV9-treated cardiomyocyte-BDNF-KO hearts (Figure 9B). In parallel, the levels of phosphorylated Smad2/3 in BDNF-AAV9-treated cardiomyocyte-BDNF-KO hearts and WT hearts were significantly lower than those in NC-AAV9-treated cardiomyocyte-BDNF-KO hearts (Figure 9C). Furthermore, WB analysis showed that the Smad7 expression level in BDNF-AAV9-treated cardiomyocyte-BDNF-KO hearts was significantly higher than that in NC-AAV9-treated cardiomyocyte-BDNF-KO hearts and WT hearts (Figure 9D). These results suggest that BDNF-AAV9-mediated ectopic BDNF gene therapy for cardiomyocyte-BDNF-KO hearts *in vivo* decreases the activity of the TGF-β/Smad2/3 pathway and the expression levels of fibrotic markers, increases Smad7 expression, and subsequently ameliorates cardiac fibrosis.

### Treatment of cardiomyocyte-BDNF-KO hearts with a BDNF mimic, 7,8-DHF, *in vivo* alters the expression levels of fibrotic markers and ameliorates cardiac fibrosis

**FIGURE 10:**
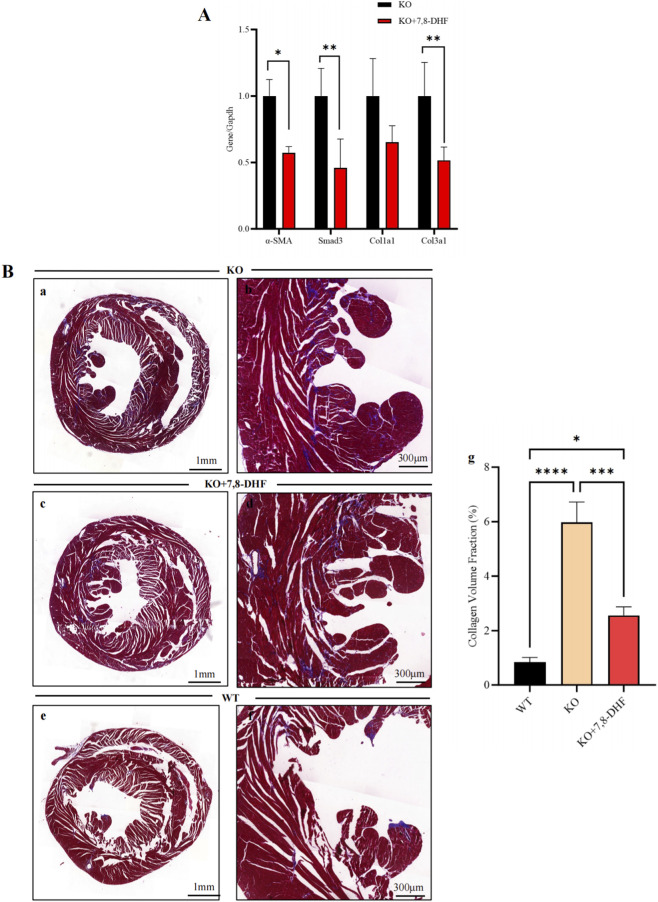
Treatment with the BDNF mimic 7,8-DHF attenuates cardiac fibrosis in cardiomyocyte-BDNF-KO hearts *in vivo*. **(A)** qPCR revealed that after 7,8-DHF treatment for 6 weeks, the expression of genes related to cardiac fibrosis mediated by the TGF-β/Smad2/3 pathway (*α-SMA, Smad3, and Col3a1*) was significantly lower in 7,8-DHF-treated cardiomyocyte-BDNF-KO hearts than in untreated cardiomyocyte-BDNF-KO hearts. **(B)** Masson’s trichrome staining showed that after treatment with 7,8-DHF for 6 weeks *in vivo*, the fibrotic area of 7,8-DHF-treated cardiomyocyte-BDNF-KO hearts was significantly smaller than that of untreated cardiomyocyte-BDNF-KO hearts, and was larger than to that of WT hearts. a, c and e: Representative images of Masson’s trichrome staining of untreated cardiomyocyte-BDNF-KO hearts, 7,8-DHF-treated cardiomyocyte-BDNF-KO hearts and WT hearts. b: Magnified image of a. d: Magnified image of c. f: Magnified image of e.g: Semiquantitative analysis of the results presented in “a, c and e”. *: *p* < 0.05. ***: *p* < 0.001. ****: *p* < 0.0001. n = 4, 3, 3 (WT, 7,8-DHF treated, and untreated groups).

The poor pharmacokinetic profile of BDNF (Agrawal et al., 2014) limits its clinical therapeutic application. Therefore, the *in vivo* therapeutic effects of 7,8-DHF (a BDNF mimic) on cardiac fibrosis were further investigated. After 7,8-DHF treatment for 6 weeks, the expression of genes related to cardiac fibrosis mediated by the TGF-β/Smad2/3 pathway (*α-SMA, Smad3,* and *Col3a1*) was significantly lower in 7,8-DHF-treated cardiomyocyte-BDNF-KO hearts than in untreated cardiomyocyte-BDNF-KO hearts. (Figure 10A). Furthermore, after 7,8-DHF treatment for 6 weeks, the fibrotic area of 7,8-DHF-treated cardiomyocyte-BDNF-KO hearts was significantly smaller than that of the untreated cardiomyocyte-BDNF-KO hearts, while it was larger than that of WT hearts (Figure 10B). 7,8-DHF therapy attenuates cardiac fibrosis in cardiomyocyte-BDNF-KO hearts *in vivo*.

## Discussion

To date, the endogenous mechanism involved in preventing and limiting the occurrence of cardiac fibrosis in the myocardium remains unclear. In the present study, through the use of snRNA-seq combined with KEGG and GO analyses as well as a cell‒cell interaction analysis, we identified that cardiomyocyte-BDNF-KO significantly increased the percentage of fibroblasts, decreased the percentage of cardiomyocytes and increased the activity of the TGF-β pathway in cardiac fibroblasts. These findings reveal the previously unknown function of cardiomyocyte-derived BDNF in limiting cardiac fibrosis by reducing the activation and proliferation of CFs and the activity of the TGF-β pathway in a single-cell scenario, suggesting a potential novel mechanism through which cardiomyocyte-derived BDNF might act as an endogenous antifibrotic regulator to prevent cardiac fibrosis by inhibiting TGF-β pathway activity and CF proliferation and activation. However, whether the BDNF–TrkB pathway works in CFs and whether this pathway engages in cross talk with the TGF-β pathway to regulate the occurrence of cardiac fibrosis are still unknown. Accordingly, the cellular and molecular mechanisms underlying the antifibrotic effects of BDNF were further investigated in the present study. We first investigated the cellular and structural basis of BDNF signal transduction in CFs. qPCR, WB and immunofluorescence staining revealed that CFs and activated CFs, CMFs express the BDNF receptor TrkB but not BDNF. C-terminal protein sequencing further confirmed that the expressed TrkB receptor is the BDNF full-length receptor TrkB-FL but not the truncated receptor TrkB-T1. The findings revealed that CFs and CMFs can receive and transduce BDNF signals through the binding of BDNF to their TrkB-FL receptors, although they do not express BDNF. In addition, the expression levels of key signaling molecules involved in the TGF-β pathway (*α-SMA, Col1a1, Col3a1, and TGF-β)* in the hearts of cardiomyocyte-BDNF-KO mice were significantly higher than those in the hearts of WT mice, which confirmed that the activity of the TGF-β pathway in CFs in the hearts of cardiomyocyte-BDNF-KO mice was increased. Furthermore, both BDNF treatment and 7,8-DHF treatment decreased the expression levels of the *TGF-β, α-SMA, Col1a1 and Col3a1* genes in the CFs of WT hearts and CMFs. Given that our study and other previous reports have demonstrated that cardiomyocytes, cardiac endothelial cells and smooth muscle cells express BDNF (Li et al., 2022; Agrawal et al., 2014; Wang et al., 2019; Cai et al., 2006), our previous report that the ablation of cardiomyocyte-derived BDNF causes cardiac fibrosis (Li et al., 2022), and in combination with the findings of the present study, we propose that cardiomyocyte-derived BDNF binds to the TrkB-FL receptor on CFs and CMFs and then transduces signals and cross-regulates the activity of the TGF-β pathway, reducing the potential of CFs and CMFs to induce cardiac fibrosis.

Accordingly, the exact function of BDNF in CFs and CMFs was further investigated. Single-cell sequencing revealed that the loss of cardiomyocyte-derived BDNF resulted in an increase in the number of CFs, suggesting that BDNF can inhibit the proliferation of CFs and CF transformation into CMFs. Therefore, the effects of BDNF on the proliferation, cell cycle, apoptosis and senescence of CFs and CMFs were evaluated. Indeed, we confirmed that both BDNF treatment and 7,8-DHF treatment inhibited the proliferation of CFs and CMFs. BDNF treatment promoted the apoptosis of CMFs, whereas 7,8-DHF treatment promoted the apoptosis of CFs and CMFs. Both BDNF treatment and 7,8-DHF treatment resulted in the accumulation of CFs and CMFs in S phase. However, neither BDNF nor 7,8-DHF treatment increased the senescence of the CFs or CMFs. These findings indicated that BDNF mediated a decrease in CF proliferation, which was caused mainly by a delay in the S phase of the cell cycle. However, BDNF mediated a decrease in CMF proliferation, which was caused mainly by a delay in the S phase of the cell cycle and the induction of apoptosis. In addition, BDNF was more likely to promote apoptosis in CMFs than in CFs. Thus, BDNF not only inhibits proliferation and cell cycling in CFs but also simultaneously affects the same functions and induces apoptosis in CMFs. This unique feature makes BDNF a natural endogenous mediator that limits the proliferation of CFs and CMFs, activates CFs, inhibits CF transformation into CMFs, and subsequently restricts cardiac fibrosis in the myocardium.

In the present study, the molecular mechanism by which the BDNF‒TrkB pathway cross-inhibits the activity of the TGF-β pathway to limit cardiac fibrosis was elucidated. We first revealed that both BDNF treatment and 7,8-DHF treatment decreased the expression of α-SMA, a key protein involved in the occurrence of cardiac fibrosis in both CFs and CMFs, suggesting that BDNF decreased cardiac fibrosis by inhibiting the activation of CFs and CF transformation into CMFs. In addition, we showed that BDNF can activate the BDNF–TrkB-FL pathway by promoting the phosphorylation of TrkB-FL in both CFs and CMFs. Importantly, the activation of the BDNF–TrkB-FL pathway in CMFs can cross-inhibit the activity of the TGF-β pathway *via* decreased phosphorylation of Smad2/3, key proteins involved in the downstream activation of the TGF-β pathway. This novel mechanism is supported by the findings that both BDNF and 7,8-DHF promoted the phosphorylation of the TrkB receptor and decreased phosphorylation of Smad2/3 in CMFs; however, in CFs, these treatments promoted only the phosphorylation of the TrkB receptor but not the phosphorylation of Smad2/3. The TGF-β/Smad2/3 pathway is key for cardiac fibrosis, and the phosphorylation of Smad2/3 is a key marker of the activated TGF-β/Smad2/3 pathway (Meng et al., 2016; Cao et al., 2012; Morikawa et al., 2016). Taken together, these findings suggest that BDNF-induced activation of TrkB signaling cross-inhibits the activity of the TGF-β/Smad2/3 pathway in CMFs, which inhibits the proliferation and activation of CFs and the transformation of CFs into CMFs and to increase the number of CFs and CMFs in the S phase of the cell cycle and the apoptosis of CMFs, ultimately contributing to limiting cardiac fibrosis. In support of these findings, in this study, we also observed that BDNF and 7,8-DHF decreased the expression levels of key signaling molecules in the TGF-β/Smad2/3 pathway *(TGF-β, α-SMA, Col1a1 and Col3a1*) in CFs and CMFs.

In addition, we also revealed that the BDNF–TrkB-FL pathway cross-inhibits the activity of the TGF-β/Smad2/3 pathway by increasing the expression of Smad7. This finding is supported by the findings of the *in vitro* and *in vivo* studies in the present study, in which both BDNF and 7,8-DHF increased the expression of Smad7, a well-known negative regulator of the TGF-β/Smad2/3 pathway (Derynck and Budi, 2019; Yan et al., 2016; Gao et al., 2022). Previous studies in the field have shown that Smad7 is associated with activated TGF-β type I receptors, which antagonize the TGF-β/Smad2/3 pathway through multiple mechanisms, including interfering with R-Smad recruitment, promoting receptor dephosphorylation, recruiting E3 ubiquitin ligases to induce receptor degradation, and blocking the functional Smad complex from interacting with DNA in the nucleus (Yan et al., 2016). Taken together, these findings suggest that the BDNF–TrkB-FL pathway cross-inhibits the activity of the TGF-β/Smad2/3/α-SMA-related cardiac fibrosis pathway by decreasing the phosphorylation of Smad2/3 and increasing Smad7 expression.

We also determined whether the mechanism identified above is also active *in vivo* in the present study. Our *in vivo* study of ectopic BDNF expression in cardiomyocyte-BDNF-KO hearts using AAV9-BDNF effectively addressed this critical issue. We found that restoring BDNF expression ameliorated cardiac fibrosis mediated by the loss of cardiomyocyte-derived BDNF, and, in parallel, decreased the expression of effector genes involved in cardiac fibrosis mediated by the TGF-β/Smad2/3 pathway (*TGF-β, α-SMA, Smad3, Cola1, Col3a1,* and *Fn-1* at the mRNA level; TGF-β, α-SMA, and Cola1 at the protein level) and the phosphorylation of Smad2/3 and increased the phosphorylation of TrkB receptors and the expression of Smad7. These results suggest that BDNF-AAV9-mediated ectopic BDNF gene therapy for cardiomyocyte-BDNF-KO hearts *in vivo* decreases the activity of the TGF-β/Smad2/3 pathway and the expression levels of cardiac fibrosis effectors, increases Smad7 expression, and subsequently attenuates cardiac fibrosis, which confirms the related findings from the snRNA-seq analysis and *in vitro* study.

In fact, our recent reports and other studies have revealed that cardiomyocyte-derived BDNF plays an important role in cardiac pathophysiology (Li et al., 2022; Yan et al., 2009; Pius-Sadowska and Machalinski, 2017; Kermani and Hempstead, 2019; Hang et al., 2021). However, we still know very little about the exact underlying cellular and molecular mechanism. In the present study, we discovered an important function of endogenous cardiomyocyte-derived BDNF and its underlying cellular and molecular mechanism, in which cardiomyocyte-derived BDNF acts as an endogenous mediator to limit the proliferation and activation of CFs and CF transformation into CMFs and to increase the accumulation of CFs and CMFs in S phase and the apoptosis of CMFs to inhibit cardiac fibrosis in the myocardium through the BDNF–TrkB-FL pathway, which cross-inhibits the activity of the TGF-β/Samd2/3/α-SMA cardiac fibrosis pathway by decreasing the phosphorylation of Smad2/3 and increasing Samd7 expression. Our previous study demonstrated that cardiomyocyte-derived BDNF plays an important role in maintaining cardiomyocyte survival and improving myocardial degeneration and cardiac regeneration (Li et al., 2022). In addition, recent reports have shown that transcription factors such as KLF1 and FOXK1, as key components of cardiac microenvironment, regulate cardiomyocyte proliferation in the adult heart and contribute to regeneration following MI (Cai et al., 2025; Hao et al., 2025; Zheng et al., 2024). Therefore, cardiomyocyte-derived BDNF may represent an important regulatory factor within the cardiac microenvironment, contributing to the control of cardiac regeneration, fibrosis, and degenerative cardiomyopathy. Further investigation of the interactions between BDNF and other key cardiac microenvironment effectors, including transcription factors may uncover novel mechanisms and therapeutic targets to enhance cardiac repair and limit cardiac fibrosis and degenerative cardiomyopathy.

However, in the clinic, cardiomyocyte-derived BDNF has been shown to function as a negative regulator of the occurrence of cardiac fibrosis, and a lack of cardiomyocyte-derived BDNF may lead to the occurrence of cardiac fibrosis, degenerative myocardial pathology and HF, which has not been widely recognized or evaluated. The findings of the present study suggest that targeting the BDNF–TrkB-FL pathway cross-inhibits the activity of the TGF-β/Smad2/3/α-SMA pathway to inhibit cardiac fibrosis, degenerative myocardial pathology and HF. Considering the poor pharmacokinetic profile of BDNF (Agrawal et al., 2014), its clinical therapeutic application has been limited. In the present study, we also evaluated the therapeutic effects of 7,8-DHF, a BDNF mimic, simultaneously. The results of the present study clearly demonstrated that the effects of the BDNF mimic 7,8-DHF are quite similar to those of BDNF *in vitro* and *in vivo*. 7,8-DHF is a polyphenolic compound present in fruits and vegetables that mimics the functions of BDNF because of its ability to bind to the BDNF receptor TrkB and induce receptor dimerization and autophosphorylation, initiating the activation of downstream signaling pathways of the BDNF/TrkB pathway (Jang et al., 2010; Chen et al., 2018; Chen et al., 2011; Zeng et al., 2012). Compared with BDNF, 7,8-DHF-induced TrkB receptor phosphorylation lasts much longer. Additionally, unlike BDNF-activated TrkB receptors, which are tagged for ubiquitination and degraded after internalization, TrkB receptors activated by 7,8-DHF are recycled to the cell surface after internalization (Liu et al., 2014). Compared with BDNF, 7,8-DHF has a longer half-life (134 min in plasma following 50 mg/kg oral administration versus less than 10 min) (Zhang et al., 2014). In addition, 7,8-DHF is orally bioactive, with an oral bioavailability of 5% (Zhang et al., 2014; Liu et al., 2016). All these merits make 7,8-DHF a highly promising treatment for clinical applications to mimic BDNF and it has good potential to treat cardiac fibrosis, degenerative cardiomyopathy and HF caused by insufficient cardiogenic BDNF.

In the present study, single-cell RNA sequencing revealed that ablation of cardiomyocytes-derived BDNF led to an increased number and proportion of endocardial cells (Figure 1F; Supplementary Table S2). Given that endocardial cells can potentially undergo transdifferentiation into mesenchymal cell types, including fibroblasts, under certain physiological and pathological conditions (Zhang et al., 2018; Han et al., 2023), this finding raises the possibility that BDNF may inhibit the proliferation of endocardial cells to regulate cardiac fibrosis. However, due to limitations in time, experiment models and technical scope, we were unable to investigate this intriguing possibility in the current study. We will address this important question in future studies.

**FIGURE 11:**
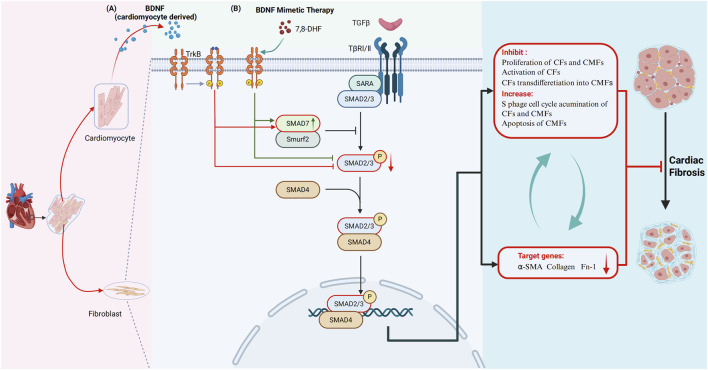
Schematic diagram of the molecular mechanism by which cardiomyocyte-derived BDNF and 7,8-DHF inhibit cardiac fibrosis. **(A)** Cardiomyocyte-derived BDNF acts as an endogenous mediator to restrict cardiac fibrosis by inhibiting CF and CMF proliferation, CF activation and transformation into CMFs and increasing the accumulation of CFs and CMFs in S phase and the apoptosis of CMFs. The BDNF‒TrkB-FL pathway cross-inhibits TGF-β/Smad2/3/α-SMA activity and increases the expression of Smad7. **(B)** Compared with BDNF, 7,8-DHF can achieve the same effects and modulate the same signaling pathways to ameliorate cardiac fibrosis. This figure was created with BioRender (https://BioRender.com).

In summary, the present study reveals a novel mechanism in which cardiomyocyte-derived BDNF acts as an endogenous mediator to limit the proliferation and activation of CFs and the transformation of CFs into CMFs and to increase the accumulation of CFs and CMFs in S phase and the apoptosis of CMFs to inhibit cardiac fibrosis in the myocardium. The above mechanism involves the BDNF–TrkB-FL pathway, which cross-inhibits the activity of the TGF-β/Smad2/3/α-SMA-related cardiac fibrosis pathway by decreasing the phosphorylation of Smad2/3 and increasing the expression of Smad7. The strategy for maintaining the appropriate level of BDNF in the myocardium and targeting the signaling pathway identified in the present study will potentially help prevent and inhibit cardiac fibrosis, degenerative cardiomyopathy and HF. 7,8-DHF is highly promising for clinical therapeutic applications because it mimics BDNF and effectively inhibits and treat cardiac fibrosis, degenerative cardiomyopathy and HF caused by insufficient amounts of cardiogenic BDNF (Figure 11).

## Data Availability

The datasets presented in this study can be found in online repositories. The names of the repository/repositories and accession number(s) can be found below: https://db.cngb.org/cnsa/, CNP0006905.
